# Data-Driven Assessment of Carbon Emission and Optimization of Carbon Emission Reduction in the Ceramic Industry

**DOI:** 10.3390/e27080872

**Published:** 2025-08-18

**Authors:** Xingbin Huang, Weihua He

**Affiliations:** School of Mathematics and Statistics, Guangdong University of Technology, Guangzhou 510520, China; 3122006381@mail2.gdut.edu.cn

**Keywords:** entropy weight TOPSIS model, XGBoost-SHAP model, multi-agent carbon trading simulation model, emission reduction strategy

## Abstract

By integrating statistical modeling and data analysis techniques, we systematically assess the carbon emission performance of the ceramic industry and propose targeted emission reduction pathways. Firstly, the entropy weight TOPSIS model is employed to quantitatively evaluate the carbon emission performance of the three major Chinese ceramic production areas: Foshan, Jingdezhen, and Zibo. Through data-driven quantitative analysis, it is disclosed that the carbon emission intensity in Foshan is significantly higher than that in the other two regions (with a relative closeness degree of 0.5185). The key issues identified include high energy consumption in the production process, a high reliance on raw coal, and insufficient investment in environmental protection. Furthermore, through the XGBoost-SHAP combined modeling, the key drivers of carbon emissions are precisely identified from multi-dimensional data. It is found that the elasticity coefficient of raw coal in the carbon emission proportion is as high as 25.84%, while the potential for substitution with natural gas is remarkable. Based on statistical prediction techniques, a carbon emission trend model under the scenario of energy structure optimization is constructed, predicting that after reaching a peak in 2017, Foshan’s carbon emissions will continue to decline, with the proportion of raw coal dropping to 48% and that of natural gas rising to 10%, thereby verifying the feasibility of the green transformation. Additionally, a multi-agent carbon trading simulation model is constructed to explore the emission reduction behaviors of enterprises under different carbon price scenarios. This study not only achieves precise quantitative analysis of carbon emissions through statistical method innovation but also verifies the feasible paths of low-carbon transformation through data modeling.

## 1. Introduction

### 1.1. Background

Ceramics, as an ancient carrier of human civilization, hold significant importance for everyone. Ceramics are a kind of hard substance made from inorganic non-metallic materials through high-temperature firing, featuring high-temperature resistance and corrosion resistance, and are widely used in home furnishing, art, industry, and other fields [1,2]. Foshan City, Guangdong Province, is a significant ceramic production base not only in China but also globally, holding a crucial position. In recent years, with the increasing national emphasis on ecological and environmental issues, the ceramic industry and its carbon emissions have drawn considerable attention. According to the available data, the ceramic industry is a high energy consumption sector, traditionally relying on fossil fuels such as coal and natural gas [3,4,5]. The energy consumption during the firing process alone accounts for over 60% of the entire production process. Each ton of ceramic products produced emits approximately 0.3 to 0.5 tons of CO_2_ [6,7,8].

According to the China County-level Carbon Emission Data Table, Foshan’s average annual carbon emissions from 2014 to 2017 were 48.77 million tons, with a peak of 49.54 million tons in 2016. In contrast, Jingdezhen, another renowned ceramic city in China, had an average annual carbon emission of only 8.28 million tons during the same period [3,4,9]. Data on Foshan’s major carbon emission sources indicate that the city’s carbon emissions mainly come from the ceramic industry, the transportation sector, and other industrial fields. Among them, the ceramic industry accounts for 30.1% of the city’s large-scale industrial carbon emissions, making it one of the primary sources of industrial carbon emissions. The main emission sources in the ceramic industry are fuel combustion and raw material calcination (CO_2_ produced from raw material decomposition) [10]. Therefore, reducing the total carbon emissions from the ceramic industry and lowering its energy and resource consumption are among the city’s top priorities [11,12,13]. The ceramic industry is in a period of industrial transformation, and the traditional model is hard to sustain. Achieving energy consumption reduction and carbon emission neutrality through technological transformation and other means is both a pressure and a blow to traditional ceramic technologies, but also an opportunity for the ceramic industry [1,2,14]. With energy substitution, technological innovation, and policy support, Foshan is expected to achieve carbon peaking, gain a competitive edge in the market, and enhance its competitiveness [11,15,16,17].

In this paper, we focus on the ceramic industry, particularly in Foshan, China. We analyze its current carbon emission status and, using existing total carbon emission data, construct multiple data-driven models to assess the emission levels. Additionally, we identify the main driving factors, simulate the carbon trading mechanism, and propose future-oriented emission reduction pathways and industrial transformation strategies [18,19].

While national and provincial CO_2_ inventory efforts by Shan et al. [3,4,20] and Xu et al. [5] have provided reliable emission baselines, their focus remains largely at macro scales. Chen et al. [21] advanced the spatial resolution by producing county-level emission datasets, identifying Foshan as a top emitter in ceramics-related CO_2_ output. However, their analysis lacked sectoral disaggregation or comparative benchmarking against other major ceramic hubs.

To address this gap, our study conducts a cross-city analysis among Foshan, Jingdezhen, and Zibo, integrating the density of ceramic enterprises with the intensity of sectoral emissions. Drawing on insights from Yu et al. [22] who explored spatial heterogeneity in emission reduction allocations at the provincial level, our work integrates spatial heterogeneity and sectoral specialization to investigate the emission intensity and enterprise distribution of the ceramic industry, thus offering a more granular emission performance diagnosis.

Carbon emissions from the ceramic industry are elevated due to high-temperature kilns, inefficient energy structures, and limited fuel substitution. Peng et al. [10] provided one of the earliest sector-specific analyses, identifying the dominance of coal and natural gas in the firing process and its resultant CO_2_ footprint. Our work builds upon such sector-specific foundations but updates the analysis with more comprehensive temporal data and a broader feature set.

We also draw on broader national studies that attribute emission shifts to industrial restructuring and technological upgrading. Guan et al. [23] and Shan et al. [4] emphasized the roles of structural transformation and energy mix optimization in driving regional emission shifts. Yet, their studies did not isolate high-emission industries like ceramics, nor did they develop predictive frameworks to inform localized mitigation strategies.

To this end, we construct a hybrid modeling system that integrates traditional multiple linear regression—commonly used in emission prediction contexts [24]—with XGBoost, a machine learning algorithm known for handling nonlinearity and interactions. Further, we employ SHAP (SHapley Additive exPlanations) for model interpretability, following the approach of Feng et al. [25]. Our use of SHAP draws inspiration from applications in landscape carbon modeling [26] and emission optimization [27], but is innovatively applied here to the ceramic manufacturing domain.

Existing literature on emission trading schemes (ETSs) in China has mostly adopted a macroeconomic lens, focusing on quota allocation fairness using game-theoretic approaches [22,28] or cost–benefit optimization under uncertainty [18]. Studies such as Tang et al. [29] and Narciso de Sousa et al. [30] employed agent-based modeling (ABM) to simulate market dynamics. However, few models simulated firm-level responses in energy-intensive industries under carbon pricing constraints.

In response, this study develops an agent-based simulation framework tailored to the ceramic manufacturing sector. Inspired by Narciso de Sousa et al. [30], who established dynamic behavioral models for agents in carbon markets, and Zhang et al. [19], who examined innovation and pricing strategies under carbon constraints, our model incorporates real enterprise emission profiles, abatement costs, and carbon price feedback.

Unlike prior studies that abstract the carbon market or generalize firm behavior, our simulation captures firm-level heterogeneity—from early adopters of clean technologies to high-emission laggards. It dynamically tracks emissions, profits, and compliance strategies under cap-and-trade scenarios, thereby revealing differentiated policy responses.

Our main contribution lies in bridging the meso-level modeling gap by developing an interpretable, industry-specific simulation framework tailored to the ceramic sector. Unlike generic macroeconomic models or narrow micro-level optimizations, our approach integrates firm behavior, regional emission characteristics, and scenario-based forecasting. This provides policymakers with a practical and scalable tool for evaluating the effectiveness of emissions trading schemes (ETSs) in high-emission industrial clusters, exemplified by Foshan City, a major hub of ceramic production in China.

### 1.2. Outline and Structure of This Paper

The structure of the remaining part of this paper is as follows: in Section 2, we introduce all the models in this paper; in Section 3, we do some experimental design based on the models and analysis the experimental results. We present a cross-regional comparative analysis of carbon emission performance in the ceramic industry using an entropy-weighted TOPSIS model (Section 3.1), in Section 3.2, we focus on the identification of key emission drivers through multiple linear regression and XGBoost, SHAP-based interpretability analysis and elasticity evaluation, and the construction of a scenario-based prediction model for carbon emission trends from 2025 to 2030 (Section 3.3); in Section 4, we conclude this paper with a summary of key findings.

## 2. Models

This section outlines the modeling frameworks employed to assess and optimize carbon emissions in ceramic industry. Three major analytical challenges are addressed using distinct yet interrelated models. Firstly, a multi-criteria decision-making model based on the entropy-weighted TOPSIS method is developed to compare regional carbon emission performance among Foshan, Jingdezhen, and Zibo. Secondly, statistical regression and machine learning models—including multiple linear regression and XGBoost—are constructed to identify the key drivers of carbon emissions and forecast future emission trends under different energy structure scenarios. Thirdly, a multi-agent simulation model is proposed to explore enterprise-level responses to carbon pricing within an Emission Trading System (ETS), combining both theoretical analysis and numerical simulation to derive optimal emission reduction strategies. Together, these models form a comprehensive framework for evaluating current emission patterns and guiding low-carbon transformation pathways.

### 2.1. Cross-Regional Comparative Analysis and Evaluation of Carbon Emission Performance

**Figure 1:**
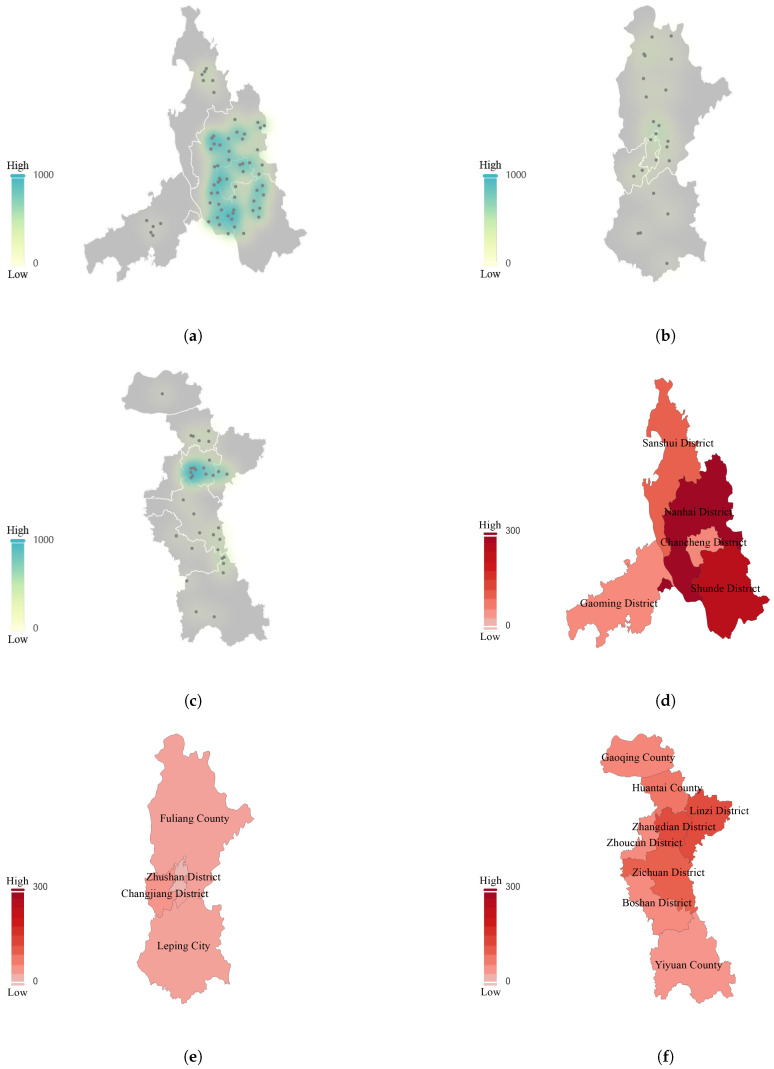
(**a**) Spatial distribution density of ceramic enterprises in Foshan, illustrating the concentration of production activities across the region. (**b**) Spatial distribution density of ceramic enterprises in Jingdezhen, highlighting key manufacturing zones and their geographical spread. (**c**) Spatial distribution density of ceramic enterprises in Zibo, showing the density of ceramic production facilities in the region. (**d**) Carbon emission heatmap of Foshan, visualizing regional carbon emission intensity and identifying high-emission areas. (**e**) Carbon emission heatmap of Jingdezhen, depicting carbon emission patterns related to ceramic production activities. (**f**) Carbon emission heatmap of Zibo, presenting the distribution of carbon emissions in relation to industrial production sites.

As the focus is on the carbon emission problem in the ceramic industry, this study utilizes data from the China Carbon Emission Accounts and Datasets (CEADs, https://ceads.net, accessed on 13 June 2025). Specifically, it analyzes the carbon emission data of 290 cities from 1997 to 2019, provided by Shan et al. [3,21], in conjunction with the number and spatial distribution density of ceramic enterprises in each region. To address the problem more effectively, the entropy weight TOPSIS model is applied in this study [31,32]. Compared to other commonly used methods such as AHP (Analytic Hierarchy Process) and DEA (Data Envelopment Analysis), the entropy-weighted TOPSIS model offers several advantages [33,34]. While AHP requires subjective expert input to assign weights, which can lead to inconsistencies, and DEA becomes computationally intensive when applied to large datasets, TOPSIS ensures objective, data-driven weight assignment through the entropy method, thus reducing bias [35,36]. Additionally, TOPSIS is computationally efficient and simple to implement, making it a better fit for handling large datasets and providing clear rankings. Moreover, while DEA is useful for efficiency evaluation, it struggles with handling multiple interdependent criteria, and AHP can become cumbersome with large numbers of criteria [37,38]. TOPSIS effectively balances both quantitative and qualitative criteria and provides a straightforward ranking of regions, which makes it particularly suitable for evaluating carbon emissions in this study. The analysis and calculations were carried out using MATLAB (R2021b) and custom-built Python (3.10.17) scripts for data processing. For detailed steps and formulas of the entropy-weighted TOPSIS method, see Appendix A. According to the statistical data of the National Bureau of Statistics, combined with the number of ceramic enterprises in Foshan, Jingdezhen, and Zibo, and other regions obtained by the enterprise data search software, the density calculation is carried out with the geographical area of each region. The distribution density of the number of ceramic enterprises in each region and the heatmap of carbon emissions in each region are shown in Figure 1.

Five indicators, ceramic enterprise density, energy consumption per unit GDP, total industrial wastewater discharge, the number of ceramic enterprises, and carbon emissions, are taken as columns to construct the decision matrix [28]. The entropy weight method and TOPSIS method are combined for application to the comprehensive evaluation of carbon emissions in various regions, and the weights are determined according to the variation degree of each attribute value. In the evaluation stage of the entropy-weighted TOPSIS model, one of the key participants is the regulatory decision-maker. This participant refers to the governmental or institutional authority responsible for formulating and enforcing carbon emission regulations, such as setting emission reduction targets, defining quota allocation rules, and monitoring compliance. Within the ceramic industry context, the regulatory decision-maker uses the TOPSIS evaluation results to assess the performance of enterprises or regions, identify priority areas for emission reduction, and guide the formulation of targeted policies and incentive measures. The evaluation model is more suitable for ceramic enterprises in various regions and carbon emissions, which also makes the influence of regional ceramic enterprises on carbon emissions more objective.

### 2.2. Identification of Carbon Emission Impact Factors and Trend Prediction

To explore how energy structure affects emissions, a multi-level model is constructed, integrating statistical and machine learning methods. In this study, carbon emission data are primarily obtained from the China Emission Accounts and Datasets (CEADs), which provide consistent city-level CO_2_ estimates based on apparent energy consumption and sector-specific emission factors. For industry-level disaggregation, we apply proportional attribution using the density and distribution of ceramic enterprises in each region, assuming a correlation between ceramic production activity and industrial emissions. The emissions considered mainly stem from fuel combustion (especially raw coal and natural gas) and raw material calcination, consistent with established practices in ceramic sector emission accounting [7,10].

This approach ensures compatibility with national emission inventories and allows integration with statistical and machine learning models. However, limitations include the possible omission of non-energy process emissions, limited resolution at the enterprise level, and a lag in data updates (latest available data is from 2021). Despite these constraints, the method offers strong consistency, scalability, and interpretability for emission trend modeling and scenario simulation.

The multiple regression linear model is a core tool in statistics for studying the linear relationship between multiple independent variables and a dependent variable. Its core idea is to quantify the independent influence of different variables on the target variable through mathematical modeling, while controlling for the interference of other variables [24]. It can clearly depict the magnitude and direction of the marginal impact of each variable on carbon emissions and conduct variable screening through coefficient significance tests (such as *p*-values and *t*-tests). Based on this model, we will conduct fitting by combining the statsmodels library. The statsmodels library is grounded in rigorous statistical theory and offers a wealth of statistical models, testing methods, and data analysis tools.

Considering that the relationship between energy structure and carbon emission may exhibit nonlinear characteristics, such as the synergy effect of the energy mix or the phenomenon of diminishing marginal returns, the XGBoost regression model (Gradient Boosting Tree) is introduced to enhance the model’s fitting accuracy and predictive ability. The XGBoost regression model is an efficient and flexible algorithm widely used in regression, classification, and ranking tasks. It can automatically model nonlinearities and variable interactions, effectively overcoming the limitations of traditional linear models. Moreover, this model is robust and suitable for small sample and high-dimensional problems.

XGBoost offers high accuracy but lacks transparency in variable interpretation. To balance model prediction performance and variable interpretability, we introduce the SHAP (SHapley Additive exPlanations) model to construct a complete XGBoost + SHAP joint analysis framework, achieving a closed loop from model prediction to interpretation [25,39]. Combined with the multiple linear regression model and the XGBoost regression model, which have good interpretability, these methods are suitable for initial variable screening and influence structure analysis [26]. The mathematical formulations and detailed explanations of the mathematical models and contribution metrics are presented in Appendix B. By integrating the multiple linear regression model and the XGBoost nonlinear regression model, we explore the modeling of carbon emission trends and scenario prediction, aiming to forecast the carbon emission changes in the ceramic industry from 2025 to 2030.

In this section, we predict the carbon emissions from 2025 to 2030 based on historical data and energy optimization scenarios. However, due to the availability of data, the most recent carbon emissions data available is up to 2021. As a result, we use the available data up to 2021 to train the model and make predictions for the years 2022 to 2030. Although we do not have actual carbon emission data for 2024, the prediction model is validated by comparing the predicted 2021 emissions with the actual emissions for that year. This comparison demonstrates the model’s feasibility and its ability to predict future emissions under different energy optimization scenarios. We will aim to update this model once the actual 2024 data becomes available.

### 2.3. Simulation of Carbon Trading Mechanism and Optimal Emission Strategy Design

As an energy-consuming manufacturing industry, the ceramic industry has a relatively high carbon emission intensity. In the short term, it is necessary to control costs and improve low-carbon and environmentally friendly technologies. In the long term, it is necessary to further improve the carbon trading mechanism.

Then we focus on the decision-making responses of typical ceramic enterprises under different carbon trading price scenarios [40]. We simulate their behavior mechanisms of emission reduction, trading, and revenue; design a reasonable simulation model; explore the relationship between enterprise emission reduction and the carbon market; and propose optimal emission reduction strategy suggestions [18,28,29].

We adopt a multi-agent interaction simulation approach, which is a method for studying the dynamics of complex systems by constructing multiple autonomous decision-making agents and their interaction rules. Meanwhile, aiming at the goal of maximizing enterprise benefits, both theoretical analysis and numerical simulation methods are employed to determine the optimal emission reduction volumes for different enterprises under various carbon trading price scenarios. Due to the different situations of various enterprises, for the sake of convenience in consideration, we will simulate three different ceramic enterprises. The detailed mathematical formulation of the multi-agent interaction simulation approach and its interpretation are provided in Appendix C.

A numerical simulation approach is employed to analyze the enterprise’s profit optimization under a given carbon price and a set of predefined parameters. Specifically, the emission reduction quantity is varied within the range [0,E] using a fixed step size. For each step, the corresponding optimal production level $x^{*}$ that maximizes the enterprise’s profit is computed. By substituting different carbon prices into the profit function, the enterprise’s profits are calculated accordingly. The optimal production strategy is then determined by identifying the value of $x^{*}$ that yields the maximum profit across the simulated range. As a result, the specific form of the enterprise’s profit function is derived and analyzed under varying carbon pricing scenarios.

## 3. Experimental Design and Analysis of the Results

### 3.1. Comparative Performance Assessment of Ceramic Industry Emissions in Foshan, Jingdezhen, and Zibo

We apply the entropy-weighted TOPSIS model to analyze the emission efficiency across three regions: Foshan, Jingdezhen, and Zibo. The five indicators of ceramic enterprise density, energy consumption per unit GDP, total industrial wastewater discharge, number of ceramic enterprises, and carbon emissions are taken as column vectors. According to the National Bureau of Statistics, China Carbon emission accounting database and RESSET macro database for data search [41,42]. The detailed construction of the decision matrix, indicator standardization process, entropy-based weight calculation, and final ranking steps are provided in Appendix D, and the results are shown in Table 1.

Through cross-regional comparison and multi-attribute decision-making model analysis, it is clearly evident that there are differences in the carbon emission influence levels of ceramic industry in Foshan City, Jingdezhen City, and Zibo City. Among them, through multi-attribute decision-making and the entropy weight TOPSIS method to evaluate and rank the performance of the carbon emission industry in the three regions, Foshan City ranks first. This indicates that under this model, the impact of ceramic enterprises on carbon emissions in Foshan City exceeds that of Zibo and Jingdezhen, and is at a relatively high level. This means that Foshan City faces greater pressure among ceramic enterprises nationwide.

### 3.2. Main Influencing Factors of Carbon Emissions

#### 3.2.1. Multiple Linear Regression Model

Firstly, we construct a multiple linear regression equation:

$$y=\beta_{0}+\beta_{1}x_{1}+\beta_{2}x_{2}+\beta_{3}x_{3}+\beta_{4}x_{4}+\varepsilon .$$

Among them, *y* represents carbon emissions, and $x_{1}$ to $x_{4}$ stand for the proportion of raw coal, petroleum, natural gas, and cement, respectively [43]. The positive and negative impacts and significance on the dependent variable are investigated; regression coefficients are visualized, a comparison chart of variable importance is constructed, and it is extended to elasticity coefficient analysis to reveal the sensitivity of carbon emissions to unit changes. All data were collected from the China Emission Accounts and Datasets (CEADs), which provides county-level CO_2_ emission inventories across China.

We use statsmodels.OLS() to fit the linear relationship between carbon emissions and the proportion of energy, output regression coefficients, t-statistics, *p*-values, $R^{2}$, and other indicators. We visualize the regression coefficients of each variable, and label the bar chart in percentage form.

**Figure 2:**
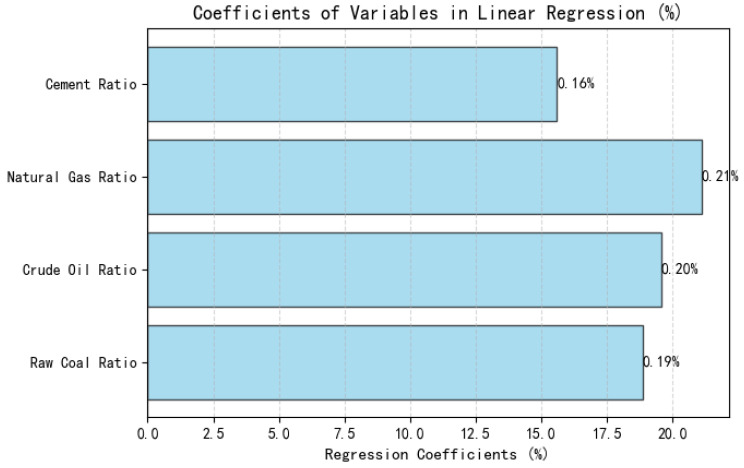
Visualization of standardized regression coefficients: impact of energy structure on carbon emissions.

The results can been found in Figure 2. It can be observed that

- The proportion of natural gas (0.21%) has the highest negative coefficient, indicating that it has significant potential to replace fossil energy and is an important driver for carbon reduction.
- The positive coefficient of petroleum (0.20%) and raw coal (0.19%) indicates that the greater their proportion in the energy structure, the stronger the “positive driving” effect on carbon emissions.
- The percentage of cement (0.16%) also shows a positive correlation, indicating that the construction materials industry, as a sector of intensive carbon, the role of industries cannot be ignored.

#### 3.2.2. Elasticity Assessment of Energy Sources on Emissions

The elasticity coefficient is given by the following:

$$\mathrm{Elasticity}\;\mathrm{coefficient}=\beta \times \left(\frac{\bar{x_{i}}}{\bar{y}}\right)$$

**Figure 3:**
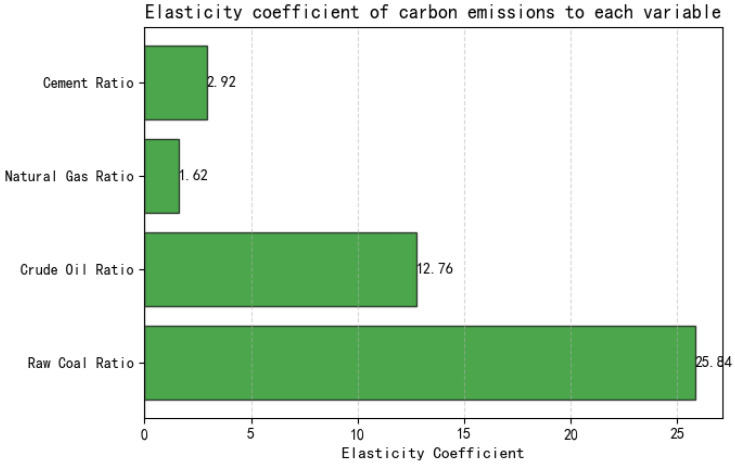
Elasticity coefficients of carbon emissions with respect to various variables.

Note that due to the small sample size in this article, the *p*-values of all variables are greater than 0.9, and the explanatory power of the model is limited. Therefore, it is necessary to make judgments in combination with the actual background. Through calculation and analysis, the elasticity coefficients of each variable have been obtained in Figure 3.

#### 3.2.3. Nonlinear Modeling and Accuracy Validation XGBoost

Then we build an XGBoost regression model. The XGBRegressor is used for training, with parameter settings of n_estimators=100 and a fixed random seed to improve reproducibility. To quantify the performance of the model in predicting carbon emissions, we compare the multiple linear regression model and the XGBoost regression model using common evaluation metrics, including the coefficient of determination ($R^{2}$), mean squared error (MSE), mean absolute error (MAE), and root mean squared error (RMSE). The results are summarized in Table 2.

It can be seen from the comparison results in the table that

- Compared with traditional linear models, the XGBoost model has a higher degree of fit ($R^{2}=0.956$), indicating that it has a stronger explanatory power.
- The MSE was reduced by over 50%, indicating a significant decrease in prediction error.
- Overall, XGBoost is a more suitable machine learning method for this problem, especially when it comes to modeling nonlinearity and variable interactions.

**Figure 4:**
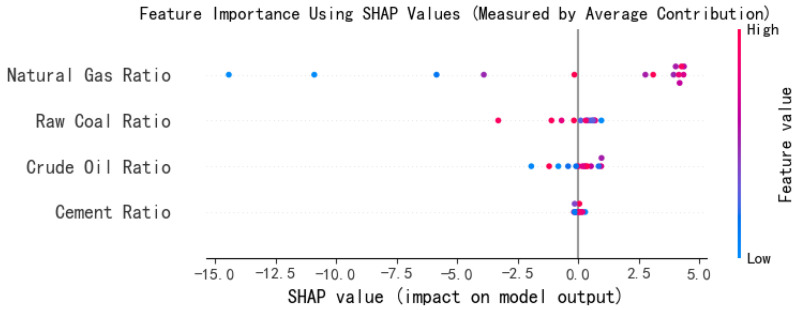
Relative importance of natural gas, raw coal, petroleum, and cement on carbon emissions.

Moreover, based on TreeExplainer, we calculate the SHAP value for each variable; the average SHAP value represents the marginal contribution or influence degree of each variable in the entire model. The mean squared error (MSE) is calculated and the result is significantly better than that of the linear model. Therefore, the SHAP value is introduced to visualize the contribution of variables, and the energy structure of raw coal, petroleum, natural gas, cement, etc., is visualized in Figure 4. This further verifies the dominant position of raw coal and natural gas.

Based on the interpretation of the SHAP plot, key insights into the relative importance and influence direction of energy-related variables on carbon emissions are derived, offering targeted guidance for policy intervention and industrial optimization. The year 2017 was selected as the base year because it represents the last stable year before significant policy changes and energy transition initiatives began to affect the carbon emission landscape. In addition, 2017 marks the peak emissions period in Foshan, providing a reliable reference point for evaluating emission reduction trends.

- Proportion of raw coal: The SHAP value is mainly concentrated in the range of 0 to 5, with high-value points mainly on the right side. This indicates that the higher the proportion of raw coal, the more significant its positive impact on carbon emissions, making it the most important positive influencing factor in the model. The red dots on the right suggest that years with a higher proportion of raw coal have a significant positive boosting effect on the predicted value of carbon emissions.
- Proportion of crude oil: The SHAP values are relatively scattered but generally lean towards the negative zone; this indicates that when the proportion of petroleum is relatively high, its driving effect on carbon emissions is not as significant as that of raw coal, and it may even have a certain negative regulatory or substitution effect. The phenomenon of blue dots being on the right and red dots on the left suggests that the influence of the variable is nonlinear or there are interaction effects.
- Proportion of cement: The distribution is relatively dense, and the SHAP values are generally close to 0; this indicates that its impact on carbon emissions is stable but limited in magnitude, and its effect is not significant.
- Proportion of natural gas: It shows a significant negative impact: when the value is high (red), the SHAP value approaches 0; when the value is low (blue), the SHAP value is significantly negative. This indicates that when natural gas replaces traditional fossil fuels, there is a clear potential for emission reduction. It is the only variable that shows an overall negative effect and has good policy promotion value.

#### 3.2.4. Simulating Future Emission Trends Under Energy Optimization Scenarios

Based on the XGBoost-SHAP model, we also combine a strategy simulation model that integrates regression prediction with energy structure constraints to conduct quantitative simulation predictions for policies such as “coal-to-gas” and “carbon neutrality pathways”.

Variable Path Function Construction: We construct the variable path function for key energy variables, specifically raw coal and natural gas. Time-varying paths are established to digitally represent policy adjustment processes.

Coal Ratio: $x_{coal}\left(t\right)=x_{coal}-\delta_{coal}\cdot \left(t-2017\right)$

Natural Gas Ratio: $x_{gas}\left(t\right)=x_{gas}-\delta_{gas}\cdot \left(t-2017\right)$

Among them, the policy adjustment amplitude is set as follows: $\delta_{coal}=1\%$, $\delta_{gas}=0.5\%$.

Additionally, the variables for petroleum and cement can be considered linearly stable or slowly decreasing. This forms a complete input variable matrix for the model.

The model prediction function is linked by using the well-trained XGBoost model as the carbon emission predictor, the corresponding energy structure variables for each year are input, and the predicted values are output:

$${\hat{y}}_{t}=f_{XGB}\left(x_{coal}\left(t\right),x_{oil}\left(t\right),x_{gas}\left(t\right),x_{cement}\left(t\right)\right).$$

**Figure 5:**
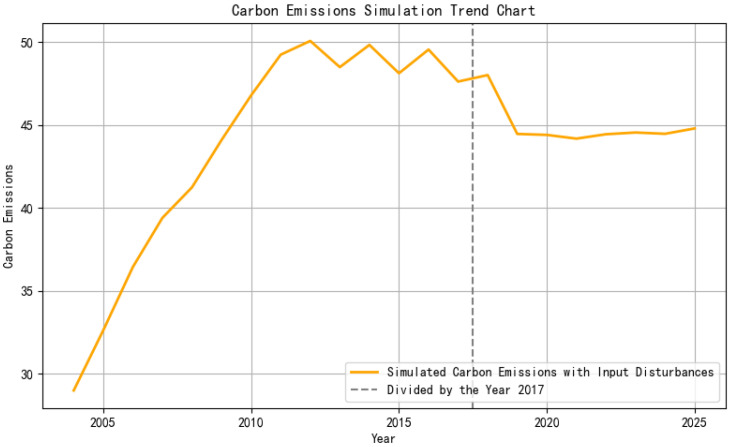
Forecast of carbon emission trends under energy structure transition scenarios.

The prediction process can be iterated on a rolling basis year by year. The time series prediction curve is given by $\left\{{\hat{y}}_{2018},{\hat{y}}_{2019},\ldots ,{\hat{y}}_{2025}\right\}$ to visually present the trend of carbon emissions. The predicted changes in carbon emissions from 2018 to 2025 can be seen in Figure 5.

### 3.3. Sensitivity Diagnostics and Dynamic Forecasting of Emission Pathways

To further examine the model’s response intensity to variations in input variables, we perturb the main energy structure variables and observe the changes in carbon emissions to analyze the model’s sensitivity and the weight of policy variables. Specifically, we record the changes in carbon emission predictions before and after disturbances, and explore the impact intensity of a single variable on carbon emissions. Each time, only one variable is adjusted individually (for example, the proportion of raw coal increases by 5%), while the rest of the variables remain unchanged. This approach allows us to isolate the effect of each individual input factor and understand its significance in influencing the predicted carbon emission levels. The analysis results are shown in Table 3.

It can be seen from the comparison results in the table that

- The proportion of raw coal is the most sensitive factor and the core driver of carbon emission changes. The +5% increase in raw coal represents a typical fluctuation observed in historical data, reflecting both changes in energy prices and patterns of industrial energy consumption. A decrease in raw coal by −5% aligns with policy-driven transitions towards cleaner energy sources and has been seen in previous reductions in coal dependency in similar industrial sectors.
- Natural gas shows a clear negative correlation with carbon emissions and is a key support target in the clean energy substitution path. The +5% increase in natural gas aligns with trends in energy optimization observed across various sectors, and a −5% decrease reflects the potential decline in natural gas usage due to shifts in energy policies or fuel costs.
- The cement industry is less affected but still cannot be ignored. The +5% increase in cement consumption is based on realistic assumptions about growing demands in the construction and industrial production sectors. The impact of cement consumption on emissions, though smaller, should be addressed alongside the broader energy conservation and emission reduction strategies in the industry.

Based on the factors that influence carbon emission collected in the ceramic industry and combined with the ceramic industry’s production process, the following conclusions are drawn: the reliance on coal remains the core challenge in carbon reduction; clean energy is a key path to reducing emissions; and multilateral collaboration is the best strategy.

### 3.4. Multi-Agent Simulation of Enterprise Emission Strategies

We set the carbon price *p* to gradually increase from USD 0 to 42, based on historical trends and projections for carbon pricing in active carbon markets, such as the European Union Emission Trading System (EU ETS). In recent years, EU ETS carbon prices have fluctuated between EUR 0 and EUR 40 per metric ton. Projections indicate that prices could reach up to EUR 149 per metric ton (USD 156/t) by 2030, driven by policy reforms and market dynamics [44]. This range was substituted into the enterprise profit formula to solve for the optimal emission reduction and the trend of profit changes for three different enterprises at different carbon prices.

**Figure 6:**
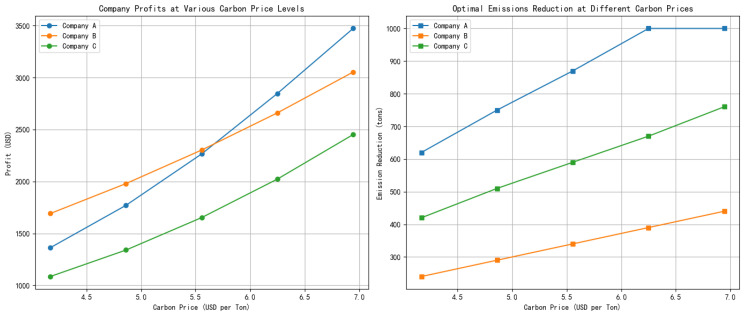
Optimal emission reduction paths of enterprises under varying carbon price levels.

After substituting the carbon price into the profit formula, the results indicate that as the carbon price increases, the optimal emission reduction amounts for all three enterprises rise. However, due to variations in emission reduction costs and technological capabilities, each enterprise exhibits different sensitivities. Figure 6 presents the simulation results for three ceramic enterprises (A, B, and C) under a carbon trading mechanism. The left panel illustrates changes in enterprise profits at various carbon price levels, while the right panel shows the corresponding optimal emission reductions. These findings highlight how carbon pricing influences enterprise behavior, balancing both economic outcomes and environmental responsibilities.

The simulation was implemented using PyCharm as the integrated development environment (IDE), where an agent-based modeling approach was employed to simulate the behavior of individual ceramic enterprises under various carbon price scenarios. Each enterprise, modeled as an agent, makes decisions based on emission reduction strategies, energy consumption patterns, and compliance with carbon pricing regulations. The simulation results track profit changes and emission reductions across different enterprise profiles, reflecting how carbon pricing influences business strategies.

Specifically, Enterprise A faces relatively high emission reduction costs and has a tight emission quota. As a result, it will only significantly reduce emissions when the carbon price surpasses USD 28. In contrast, Enterprise B has the lowest emission reduction costs and advanced technological capabilities, motivating it to reduce emissions even at relatively low carbon prices, reflecting an active approach to emission reduction. Enterprise C, with intermediate characteristics, shows a stable and consistent response to emission reduction, demonstrating a more moderate approach to addressing carbon pricing.

**Figure 7:**
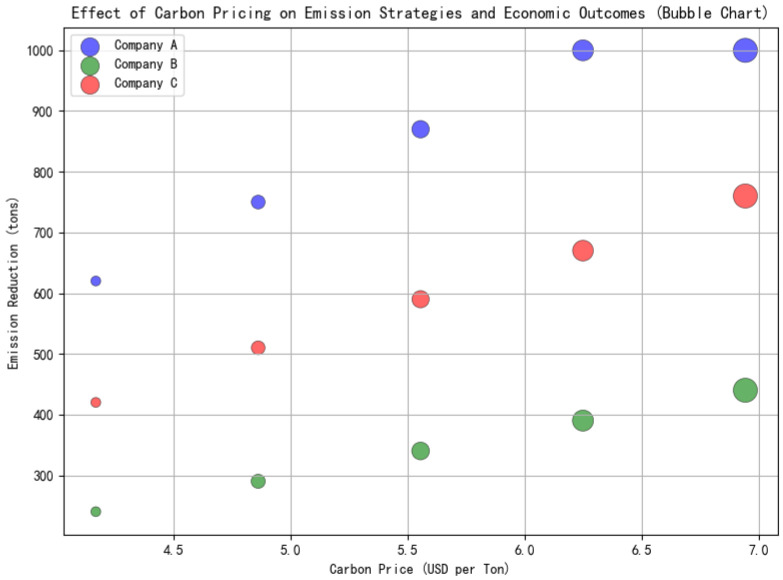
Trends of profit changes in different enterprises.

Based on the enterprise profit function, Figure 7 illustrates how optimal emission reduction levels vary with increasing carbon prices across different types of ceramic enterprises. As the carbon price rises, the emission reduction efforts increase, though the sensitivity and response thresholds differ by enterprise type.

As shown in Figure 7, the following key insights can be drawn:

- When the carbon price of an enterprise is low, implementing emission reduction measures may lead to a decline in the enterprise’s profits.
- In the medium to high range of carbon prices, reasonable emission reduction not only reduces the expenditure on quota purchases but also enhances overall profits.
- There is a distinct “critical carbon price point” in the graph. Once exceeded, enterprises’ willingness to reduce emissions increases significantly.

**Figure 8:**
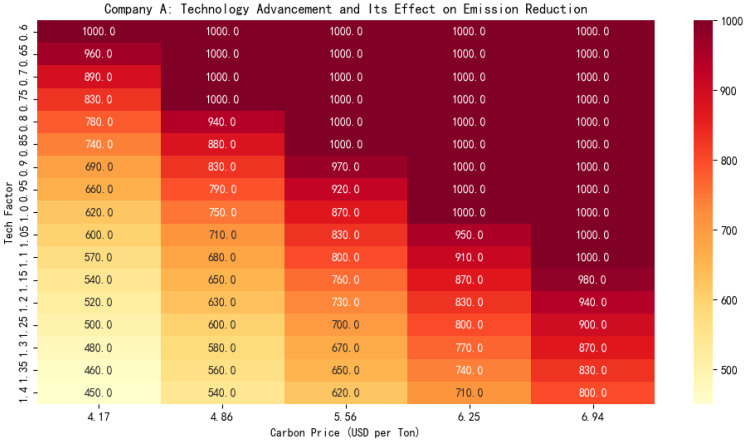
Heatmap of optimal emission reduction under combined effects of technological progress and carbon pricing.

We also draw the emission reduction heatmap as shown in Figure 8. It illustrates the interaction between carbon pricing and technological progress on enterprise-level emission reduction behaviors. The heatmap visualizes how different combinations of carbon price levels and technological advancements influence the optimal amount of emissions reduction. Key observations include the following:

- For enterprises, as the carbon price rises, the amount of emissions reduction will increase.
- Technological innovation and progress, along with rapid and efficient technological advancement, will bring about greater emission reduction effects for enterprises.
- There is a mutually reinforcing amplification effect between carbon prices and technology.

**Figure 9:**
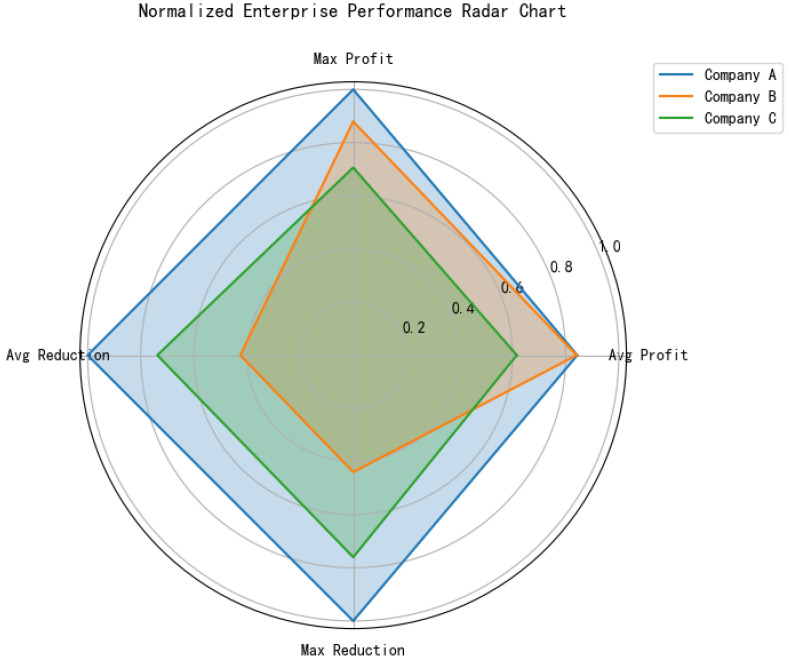
Radar chart of the normalized comprehensive performance of representative enterprises.

The comprehensive performance radar chart is generated to deeply explore the distinctive features of the three enterprises in Figure 9. By comparing the normalized performance of the three enterprises in four dimensions: average profit, maximum profit, average emission reduction, and maximum emission reduction, a comprehensive performance radar chart of the enterprises is created. Figure 9 presents that Company A faces the greatest pressure to reduce emissions and relies on high carbon prices for incentives; Company B has the best overall performance, the strongest flexibility in emission reduction and a relatively high profit level; and Company C has an average overall performance and is balanced and stable.

## 4. Conclusions

In this paper, we present an integrated modeling framework to guide the sustainable transformation of the ceramic industry. By combining classical statistical models, machine learning algorithms, and simulation techniques, we provide both explanatory insights and predictive power to support policy formulation and enterprise-level decision-making under the dual-carbon goals. The proposed methodology incorporates four key components:

- The TOPSIS-entropy weight method, which enhances objectivity and robustness of the multi-criteria decision-making process by combining entropy-based weighting with proximity-based ranking mechanisms.
- The multiple linear regression model, which elucidates the relationships between the variables of the energy structure and carbon emissions. It offers interpretable coefficients that identify key emission-driving energy sources and provide an empirical basis for the targeted substitution of clean energy.
- The XGBoost nonlinear regression model, combined with SHAP explainability tools. It allows for high-precision prediction and dynamic simulation of carbon emission trends, uncovering nonlinear influences, and enabling scenario-based policy analysis.
- The multi-agent interaction simulation, which constructs autonomous decision-making entities to simulate carbon trading dynamics and optimize emission reduction strategies. This component facilitates a more intuitive understanding of heterogeneous enterprise behaviors, and provides data support and theoretical basis for the government to formulate policies and for enterprises to achieve optimal emission reduction.

Empirical results identify the high-temperature firing stage as the main emission source in ceramic production. The use of raw coal shows a significant positive effect on emissions, while natural gas demonstrates a mitigating effect. In addition, simulation analyzes reveal that carbon pricing serves as a key driver of corporate emission reduction behaviors. A distinct threshold effect is observed: Below a certain price point, firms demonstrate a limited incentive to reduce emissions; beyond this point, their abatement efforts increase markedly. This highlights the strategic importance of well-calibrated, market-based policy instruments.

Current frameworks could be improved by incorporating additional explanatory variables (e.g., GDP growth, energy prices, carbon sequestration dynamics) and enhancing algorithmic performance [45]. Furthermore, future research should integrate causal inference frameworks to delineate the directionality and magnitude of variable interactions, while dynamic optimization algorithms—such as reinforcement learning and optimal control models—could be employed to derive cost-effective, adaptive emission reduction pathways.

Overall, this integrated modeling approach provides a robust analytical foundation for the green transformation of the ceramic industry. It provides actionable insights for companies seeking to balance environmental and economic objectives and equips policymakers with rigorous evidence to support science-based, innovation-led carbon governance. The approach also has the potential for broader application in other resource-intensive sectors, contributing significantly to national and global targets of carbon neutrality and sustainable industrial transformation.

## Figures and Tables

**Table 1:** Evaluation ranking of carbon emission performance in three ceramic industry regions.

Region	Carbon Emission Performance	Rank
Foshan	0.5185	1
Zibo	0.2755	2
Jingdezhen	0.2060	3

**Table 2:** Comparison results between linear regression model and XGBoost regression model.

Model Type	$R^{2}$	MSE	MAE	RMSE
Multiple Linear Regression	0.878	15.26	3.10	3.91
XGBoost Regression Model	0.956	6.88	2.04	2.62

**Table 3:** Carbon emission forecast table.

Energy Structure	Adjustment Range	Emission Change (100 Million Tons)	Relative Change Rate
Raw Coal (Up)	+5%	+2.31	+4.1%
Raw Coal (Down)	−5%	−2.45	−4.4%
Natural Gas (Up)	+5%	−1.12	−2.0%
Natural Gas (Down)	−5%	+1.26	+2.2%
Cement (Up)	+5%	+0.67	+1.1%

## Data Availability

All data can be accessed from the CEADs platform: https://www.ceads.net/data/province/, https://data.stats.gov.cn and https://www.resset.cn, accessed on 13 June 2025.

## Appendix A. Entropy-Weighted TOPSIS Method

This appendix provides the detailed steps and formulas used in the entropy-weighted TOPSIS method for evaluating carbon emissions.

### Appendix A.1. Construction of Decision Matrix and Normalization

Let $X={\left[x_{ij}\right]}_{m\times n}$ be the decision matrix, where *m* alternatives are evaluated across *n* criteria. Normalization is conducted based on indicator type:

- For the “smaller-the-better” indicators:
  (A1) $$x_{ij}^{\prime }=\max\left(x_{j}\right)-x_{ij}$$
- For point-optimal indicators (with optimal point *a*):
  (A2) $$x_{ij}^{\prime }=1-\frac{|x_{ij}-a|}{\max(|x_{j}-a|)}$$
- For the “larger-the-better” indicators:
  (A3) $$x_{ij}^{\prime }=x_{ij}$$

### Appendix A.2. Entropy-Based Weight Calculation

The proportion of the *i*-th alternative under the *j*-th criterion is as follows:

(A4) $$P_{ij}=\frac{r_{ij}}{\sum_{i=1}^{m}r_{ij}}$$

The entropy for each criterion is as follows:

(A5) $$E_{j}=-\frac{1}{\ln m}\sum_{i=1}^{m}p_{ij}\ln p_{ij}$$

The corresponding weight is then derived as follows:

(A6) $$\omega_{j}=\frac{1-E_{j}}{\sum_{j=1}^{n}\left(1-E_{j}\right)}$$

### Appendix A.3. Weighted Normalized Matrix

Define the enhanced normalized matrix as follows:

(A7) $$r_{ij}^{\prime }=\frac{r_{ij}}{\sqrt{\sum_{i=1}^{m}r_{ij}^{2}}}$$

The weighted matrix is then given by the following:

(A8) $$v_{ij}=r_{ij}^{\prime }\cdot \omega_{j}$$

### Appendix A.4. Calculation of Ideal Solutions and Relative Closeness

The positive ideal solution (denoted as $v^{+}$) and the negative ideal solution (denoted as $v^{-}$) are, respectively, composed of the maximum and minimum elements of each column vector in *V*. For each scheme $A_{i}$, the Euclidean distance between scheme $A_{i}$ and the positive ideal solution is calculated as follows:

(A9) $$S_{i}^{+}=\sqrt{\sum_{j=1}^{n}{\left(v_{ij}-v_{j}^{+}\right)}^{2}}$$

where $v_{j}^{+}$ is the *j*-th component of $v^{+}$, resulting in $S^{+}={\left(S_{i}^{+}\right)}^{\left(j\right)}$.

The Euclidean distance between $A_{i}$ and the negative ideal solution is calculated as follows:

(A10) $$S_{i}^{-}=\sqrt{\sum_{j=1}^{n}{\left(v_{ij}-v_{j}^{-}\right)}^{2}}$$

where $v_{j}^{-}$ is the *j*-th component of $v^{-}$, resulting in $S^{-}={\left(S_{i}^{-}\right)}^{\left(j\right)}$.

Relative closeness: Define the relative closeness of scheme $A_{i}$ to the positive ideal solution as follows:

(A11) $$c_{i}^{+}=\frac{s_{i}^{-}}{s_{i}^{+}+s_{i}^{-}},\;0<c_{i}^{+}<1$$

Calculate $C^{+}={\left(C_{i}^{+}\right)}^{\left(j\right)}$, and after normalization, obtain the optimal ranking of all schemes.

## Appendix B. Multiple Linear Regression Model and XGBoost Regression Model

### Appendix B.1. Multiple Linear Regression Model

The basic form of the multiple linear regression model is defined as follows:

(A12) $$y=\beta_{0}+\beta_{1}x_{1}+\beta_{2}x_{2}+\ldots +\beta_{n}x_{n}+\varepsilon$$

Explanation of the terms:

- *y*: Dependent variable, the predicted variable of the model.
- $x_{1},x_{2},\ldots ,x_{n}$: Independent variables, used to explain or predict the dependent variable. These can be continuous or discrete.
- $\beta_{0}$: Intercept term, the expected value of *y* when all $x_{i}$ are 0.
- $\beta_{1},\beta_{2},\ldots ,\beta_{n}$: Regression coefficients, representing the influence of each $x_{i}$ on *y*.
- ε: Error term, capturing unobserved variation, assumed to follow a normal distribution with mean 0.

### Appendix B.2. XGBoost Regression Model

The expression for the XGBoost-based contribution coefficient is as follows:

(A13) $$E_{i}=\frac{\beta_{i}\cdot \bar{x_{i}}}{\bar{y}}$$

Explanation of the terms:

- $\beta_{i}$: Regression coefficient for the *i*-th independent variable.
- $\bar{x_{i}}$: Mean of the independent variable $x_{i}$.
- $\bar{y}$: Mean of the dependent variable *y*.

This coefficient reflects the expected percentage change in *y* (e.g., carbon emissions) for each 1% increase in $x_{i}$, providing a basis for adjusting policy direction and intensity.

## Appendix C. The Multi-Agent Interaction Simulation Approach

To evaluate the optimal emission reduction strategy for ceramic enterprises, a profit maximization model is developed based on carbon trading mechanisms and enterprise-level parameters. The analysis integrates both theoretical derivation and numerical simulation.

### Appendix C.1. Model Assumptions and Parameters

Let the following parameters be defined as follows:

- *E*: Baseline carbon emissions (before reduction);
- *A*: Carbon quota allocated to the enterprise;
- *u*: Unit production profit (sales revenue per emission unit);
- α: Emission reduction cost coefficient;
- $f_{\mathrm{tech}}$: Technology factor influencing reduction cost;
- *p*: Carbon trading price, with p∈(0,42).

The emission reduction cost function is defined as follows:

(A14) $$C\left(x\right)=\alpha x^{2}\cdot f_{\mathrm{tech}}$$

The actual emission amount is calculated as follows:

(A15) R=E−x

According to the carbon market mechanism:

- If R<A, the enterprise generates surplus carbon credits and can sell A−R units at market price;
- If R>A, the enterprise must purchase R−A additional quotas, incurring compliance costs.

Hence, the total profit function of the enterprise is expressed as follows:

(A16) $$\pi \left(x\right)=u\left(E-x\right)-\alpha x^{2}\cdot f_{\mathrm{tech}}+p\left(A-\left(E-x\right)\right).$$

This function comprises the following components:

- u(E−x): Revenue from sales, assumed to be proportional to emissions;
- $\alpha x^{2}\cdot f_{\mathrm{tech}}$: Cost of emission reduction;
- p(A−(E−x)): Net revenue (or cost) from carbon trading activities.

Simplifying:

(A17) $$\pi \left(x\right)=\left(u+p\right)\left(E-x\right)-\alpha x^{2}\cdot f_{\mathrm{tech}}+pA$$

### Appendix C.2. Theoretical Derivation of the Optimal Solution

Take the first derivative as follows:

(A18) $$\frac{d\pi }{dx}=-\left(u+p\right)-2\alpha xf_{\mathrm{tech}}$$

Set the derivative equal to zero:

(A19) $$\frac{d\pi }{dx}=0\Rightarrow -\left(u+p\right)-2\alpha xf_{\mathrm{tech}}=0$$

Solving for *x*,

(A20) $$x^{*}=\frac{-(u+p)}{2\alpha f_{\mathrm{tech}}}$$

Since u>0, p>0, α>0, and $f_{\mathrm{tech}}>0$, it follows that $x^{*}<0$, which is not feasible under the constraint x∈[0,E].

Accordingly, we consider the boundary conditions to determine the optimal solution as follows:

(A21) π(0)=uE+p(A−E)

(A22) $$\pi \left(E\right)=-\alpha E^{2}f_{\mathrm{tech}}+pA$$

Thus, the optimal solution under theoretical analysis is achieved at x=0 or x=E depending on which boundary yields a higher profit.

### Appendix C.3. Carbon Trading Incentive Analysis

The revenue from carbon allowance trading is as follows:

(A23) p(A−(E−x))=p(x+A−E)

When x>E−A, the enterprise earns positive revenue from selling surplus allowances, potentially offsetting the cost of reduction and encouraging further emission reduction.

### Appendix C.4. Numerical Simulation Approach

To verify the analytical conclusion, a numerical simulation is implemented. Given fixed enterprise parameters and varying carbon prices within (0,42), the emission reduction quantity *x* is incrementally adjusted within the domain [0,E]. At each discrete point, the corresponding profit π(x) is computed. The final optimal emission reduction level is thus determined as follows:

(A24) $$x_{\mathrm{final}}^{*}=\arg\max_{x\in [0,E]}\pi \left(x\right).$$

This modeling approach facilitates an in-depth evaluation of enterprise behavior under carbon market incentives and provides policy insights for promoting emission abatement through economic instruments.

## Appendix D. TOPSIS-Based Performance Evaluation Calculations

This appendix presents the detailed computational steps for the entropy-weighted TOPSIS model used to assess regional carbon emission performance across Foshan, Jingdezhen, and Zibo.

### Appendix D.1. Indicator Definition and Data Clarification

The five selected indicators represent the characteristics of the intensity of the environmental and industrial environment of each region.

- I: Ceramic Enterprise Density (units/km^2^)—Reflects the spatial clustering of ceramic production.
- II: Energy Consumption per Unit GDP (tce/10^4^ CNY)—Indicates energy efficiency of the regional economy.
- III: Total Industrial Wastewater Discharge (10^4^ tons)—Represents general pollutant output.
- IV: Number of Ceramic Enterprises—A proxy for regional ceramic industry scale.
- V: Total Carbon Emissions (10^4^ tons)—The main variable of interest for emission performance.

All data are obtained from authoritative sources including the National Bureau of Statistics, the China Carbon Emission Database, and the RESSET macroeconomic database, with values taken as the most recent available for a consistent year across the three regions.

### Appendix D.2. Decision-Making Roles

In this evaluation framework, two decision-making roles are modeled as follows:

- Weighting Stage: The entropy method acts as an objective evaluator, assigning weights based on the degree of dispersion in each indicator across alternatives.
- Evaluation Stage: A regulatory decision-maker (e.g., local environmental authority) uses the TOPSIS ranking to assess enterprise performance and prioritize emission control strategies.

### Appendix D.3. TOPSIS Computational Process

#### Appendix D.3.1. Decision Matrix Construction

Let $x_{ij}$ denote the original value of indicator *j* for region *i*. The initial decision matrix is shown in Table A1.

**Table A1:** Decision matrix of regional indicators.

No.	I	II	III	IV	V
A	1.72	0.50	26,683	6519	47.61
B	0.48	0.65	5296	2516	8.85
C	0.64	0.58	21,212	3828	41.43

All five indicators are of the “smaller-the-better” type. The decision matrix is positively transformed by the following:

$$x_{ij}^{\prime }=\max\left(x_{ij}\right)-x_{ij},\;\mathrm{resulting}\;\mathrm{in}\;\mathrm{transformed}\;\mathrm{matrix}\;X_{1}.$$

#### Appendix D.3.2. Evaluation Steps

1. Normalize the data using the standard method:
  $$X_{2}={\left(r_{ij}\right)}_{m\times n}$$
2. Entropy method yields the entropy values:
  $$E_{j}=\left[0.3348,0.5928,0.6252,0.5143,0.6314\right]$$
3. The computed indicator weights are as follows:
  $$\omega_{j}=\left[0.2890,0.1769,0.1629,0.2110,0.1602\right]$$
4. Multiply the standardized decision matrix data $r_{ij}^{\prime }$ to form matrix *V*.
5. Calculate the Euclidean distance to ideal solutions:
  $$S_{i}^{+}=\left[0.1559,0.3925,0.2944\right],\;S_{i}^{-}=\left[0.3925,0.1559,0.1808\right]$$
6. Compute relative closeness:
  $$C^{+}=\left[0.7157,0.2843,0.3804\right],\;\mathrm{with}\;0<C_{i}<1$$
